# DDoS classification of network traffic in software defined networking SDN using a hybrid convolutional and gated recurrent neural network

**DOI:** 10.1038/s41598-025-13754-1

**Published:** 2025-08-09

**Authors:** Ahmed M. Elshewey, Safia Abbas, Ahmed M. Osman, Eman Abdullah Aldakheel, Yasser Fouad

**Affiliations:** 1 Department of Computer Science, Faculty of Computers and Information, Suez University, P.O.Box:43221, Suez, Egypt; 2 Department of Computer Science, Faculty of Computer and Information Sciences, Ain Shams University, Cairo, Egypt; 3 Department of Computer Science, the Higher Future institute for Specialized Technological Studies, Cairo, Egypt; 4 Department of Information Systems, Faculty of Computers and Information, Suez University, P.O.Box:43221, Suez, Egypt; 5 Department of Computer Sciences, College of Computer and Information Sciences, Princess Nourah Bint Abdulrahman University, 11671 Riyadh, Saudi Arabia

**Keywords:** DDoS detection, CNN, GRU, Network traffic classification, Deep neural networks, Software-Defined networking, Convolutional and gated recurrent neural network, Electrical and electronic engineering, Computer science, Information technology

## Abstract

Deep learning (DL) has emerged as a powerful tool for intelligent cyberattack detection, especially Distributed Denial-of-Service (DDoS) in Software-Defined Networking (SDN), where rapid and accurate traffic classification is essential for ensuring security. This paper presents a comprehensive evaluation of six deep learning models (Multilayer Perceptron (MLP), one-dimensional Convolutional Neural Network (1D-CNN), Long Short-Term Memory (LSTM), Gated Recurrent Unit (GRU), Recurrent Neural Network (RNN), and a proposed hybrid CNN-GRU model) for binary classification of network traffic into benign or attack classes. The experiments were conducted on an SDN traffic dataset initially exhibiting class imbalance. To address this, Synthetic Minority Over-sampling Technique (SMOTE) was applied, resulting in a balanced dataset of 24,500 samples (12,250 benign and 12,250 attacks). A robust preprocessing pipeline followed, including missing value verification (no missing values were found), feature normalization using StandardScaler to standardize numerical values, reshaping the data into 3D format to fit temporal models like CNN and GRU, and stratified train-test split (80% training, 20% testing) to maintain class distribution. The CNN-GRU model integrates a 1D convolutional layer for spatial pattern extraction and a GRU layer for temporal sequence learning, followed by dense layers with dropout regularization. The model was trained using the Adam optimizer with early stopping to prevent overfitting. Among all models, the CNN-GRU hybrid achieved perfect test performance, with 100% accuracy, 1.0000 precision, recall, and F1-score, and an ROC AUC of 1.0000. It also demonstrated exceptional generalization, achieving a mean cross-validation (CV) accuracy of 99.70% ± 0.09% and a mean AUC of 1.0000 ± 0.0000 across 5-fold stratified cross-validation. While individual models such as GRU, 1D-CNN, and LSTM also showed strong performance, the CNN-GRU hybrid consistently outperformed them in both accuracy and stability. These results validate the effectiveness of combining convolutional and recurrent architectures, augmented with data balancing via SMOTE, for highly accurate SDN-based intrusion detection.

**Supplementary Information:**

The online version contains supplementary material available at 10.1038/s41598-025-13754-1.

## Introduction

With the increasing reliance on information and communication technology (ICT) systems across industries and daily life, ensuring the security of these infrastructures has become a critical challenge^1,2^. As cyber threats become more sophisticated and frequent, robust network protection mechanisms are essential. Among these, intrusion detection (ID) systems have emerged as key tools for identifying and mitigating network-based attacks. Initially conceptualized in the 1980s^3^, ID systems have since developed into essential components of cybersecurity frameworks, capable of analyzing both internal and external traffic to detect suspicious activities. They are typically classified into network-based, host-based, and hybrid systems, each offering varying degrees of visibility and protection depending on the architecture and application. The integration of these systems within modern network environments, especially those based on SDN, is vital for real-time threat detection and response^4,5^.

As SDN continues to be widely adopted for its flexibility and centralized control, it has simultaneously become an attractive target for DDoS attacks due to its centralized architecture and programmability. Traditional IDS often struggle to accurately detect sophisticated or evolving DDoS patterns in real time, particularly in dynamic SDN environments. Moreover, existing detection models may suffer from high false positive rates, poor generalization to unseen attack types, and inadequate handling of temporal and spatial features within network traffic^6,7^. This creates a critical need for intelligent, robust, and high-performing intrusion detection frameworks capable of real-time traffic classification and threat identification. Therefore, this paper addresses the problem by proposing a hybrid model that combines CNN for spatial feature extraction with GRU for temporal pattern recognition, aiming to enhance accuracy, recall, and AUC in detecting DDoS attacks.

Recent advancements have introduced deep hybrid and attention-based models for DDoS detection in SDN environments, such as transformer-enhanced frameworks, residual GRU networks, and attention-weighted CNN-LSTM architectures^9,14,17^. These models demonstrate a trend toward integrating spatial, temporal, and contextual awareness in intrusion detection systems.

In this context, the hybrid CNN-GRU architecture is particularly well-suited for the DDoS detection task. CNN layers effectively extract spatial patterns from traffic features such as flow size and packet rates, while GRU layers capture temporal dependencies across sequences of network behavior, allowing the model to detect both short-term anomalies and long-term attack trends. Compared to standalone models like LSTM or CNN, the CNN-GRU architecture offers a balanced trade-off between accuracy, training speed, and memory efficiency, making it practical for deployment in real-time SDN-based systems.

The main target of this paper is to develop and evaluate an intelligent hybrid DL model for the accurate detection of DDoS attacks in SDN environments. To achieve this, the paper pursues the following specific objectives:

1. balance SDN network traffic using normalization techniques and SMOTE to ensure fair and reliable model training.
2. design and implement a hybrid deep learning architecture that integrates CNN and GRU for capturing temporal dependencies in network traffic.
3. evaluate the CNN-GRU model against other standalone deep learning models such as MLP, 1D-CNN, LSTM, GRU, and RNN in terms of accuracy, precision, recall, F1-score, and ROC AUC.
4. validate the robustness and generalization capability of the CNN-GRU model using fivefold cross-validation and test set analysis.
5. demonstrate the feasibility of the proposed model for real-time and scalable intrusion detection in SDN environments, thereby enhancing network security and resilience.

This research makes several contributions to the field of network security and DL-based ID:

1. A novel hybrid CNN-GRU architecture is proposed for DDoS attack detection in SDN environments, leveraging the strengths of CNN for spatial feature extraction and GRU for modeling dependencies in traffic patterns.
2. An effective data preprocessing pipeline is implemented, including normalization, reshaping, and class balancing using SMOTE, to improve model training reliability and mitigate the impact of class imbalance.
3. Extensive experiments using fivefold cross-validation and test set evaluation are performed to validate the stability, generalization, and robustness of the CNN-GRU model in real-world SDN scenarios.
4. The proposed CNN-GRU model achieves superior performance, with perfect detection metrics (100% accuracy, 1.0000 precision, recall, F1-score, and AUC), demonstrating its practical viability for real-time, scalable, and high-precision intrusion detection in modern SDN infrastructures.

The hybridization of CNN and GRU provides an effective architectural synergy for detecting DDoS attacks in SDN environments. CNN layers are particularly adept at extracting spatial or local features from network traffic data. They operate by convolving filters over input sequences, enabling the identification of significant local patterns such as abnormal packet size distributions or flow durations. These patterns are often indicative of DDoS behavior, especially when such anomalies occur in bursts. By capturing localized dependencies and reducing input dimensionality while preserving essential information, CNNs serve as powerful feature extractors for network intrusion tasks.

The remainder of this paper is organized as follows:

- Section “Related works” reviewing existing intrusion detection techniques in SDN and recent advances in deep learning-based DDoS detection methods.
- Section “Methodology” describes the methodology besides dataset, preprocessing steps and outlines the experimental setup / details the architecture of the proposed hybrid CNN-GRU model.
- Section “Results and discussion” presents the experimental results, cross-validation outcomes, and performance comparison of all models.
- Section “Conclusion and future work” mentions the conclusion and future research.

## Related works

The following section reviews recent and relevant works on intrusion detection in SDN using deep learning approaches, highlighting their methodologies, datasets, performance, and limitations. This review provides a foundation for the proposed hybrid CNN-GRU model and positions it within the current research landscape.

Yousuf and Mir^8^ introduced DALCNN, a DDoS detection algorithm for IoT networks leveraging an RNN-based model integrated into a SDN environment. Their solution employed a three-tier architecture with a novel activation function to enhance classification accuracy. Tested on 177 attack instances using Mininet and Wireshark, the model achieved a remarkable 99.98% accuracy and nearly perfect scores across all key evaluation metrics. Furthermore, they conducted a comparative performance analysis of popular SDN controllers, concluding that OpenDayLight delivered superior results in throughput, latency, and controller responsiveness.

Wang et al.^9^ addressed the complexity of DDoS detection in SDN by proposing ARSAE-QGRU, a deep hybrid model that integrates attention mechanisms and residual connections into a stacked autoencoder architecture. These enhancements enable the model to preserve and propagate critical information during training, while the addition of a GRU component improves the model’s ability to learn temporal dependencies in traffic patterns. The model achieved accuracy rates up to 99.8% in SDN environments and also performed strongly on benchmark datasets like CICIDS2017 and CICDDoS2019, demonstrating its robustness in handling high-dimensional and temporally complex traffic data.

Wahab et al.^10^ introduced SDN-enabled hybrid DL framework for ID in IoT-based environments. The system leverages cognitive intelligence to interpret and react to traffic patterns while being optimized for lightweight deployment on resource-constrained IoT devices. The model achieved high classification accuracy (99.86%), outperforming previous hybrid models like Cu-GRU + LSTM and Cu-GRU + DNN. This work demonstrates the potential of integrating cognitive computing and SDN with deep learning to enhance IoT security without overwhelming device resources.

Razib et al.^11^ presented DNNLSTM, a DL-based SDN for securing IoT networks against both frequent and rare cyber threats. Their model combines the capabilities of deep neural networks with LSTM units to capture complex temporal patterns in network traffic. Trained on the CICIDS2018 dataset, the model achieved strong performance, with an accuracy of 99.55% , outperforming baseline models like DNNGRU and BLSTM as well as several existing solutions from prior studies. This work demonstrates the value of combining SDN and deep sequence learning techniques for scalable and intelligent IoT threat detection.

Javeed et al.^12^ proposed an efficient SDN-integrated DL framework for threats detection in IoT networks. Their model leverages CUDA-accelerated neural network architectures, including Cu-DNNGRU and Cu-BLSTM, to ensure both accuracy and computational efficiency. Trained on the CICIDS2018 dataset and validated using tenfold CV, the model achieved 99.87% accuracy. Compared to other hybrid architectures such as Cu-GRULSTM and Cu-DNNLSTM.

Alghazzawi et al.^13^ proposed a CNN-BiLSTM model for DDoS detection, aiming to overcome the shortcomings of traditional ML/DL methods that often struggle with suboptimal feature selection and neglect sequence information. Their model integrates convolutional layers to extract spatial features and BiLSTM layers to retain bidirectional temporal context. The approach employs a feature ranking technique to select the most informative inputs from the CIC-DDoS2019 dataset. The model achieved 94.52% accuracy, showing improved performance over standalone classifiers and traditional feature encoding approaches in detecting DDoS attacks.

Cui et al.^14^ introduced CNNA-BiLSTM, a hybrid DL model that combines CNN and attention-enhanced bidirectional LSTM for multi-class intrusion detection in SDN environments. Addressing the shortcomings of existing DDoS-focused detection systems, their model incorporates a feature selection mechanism to reduce dimensionality and focus on high-impact attributes. It is designed to classify eight types of attacks from the InSDN dataset, and achieved 99.86% accuracy in binary classification and 99.31% in multi-class scenarios. Notably, CNNA-BiLSTM outperformed standard models in detecting rare attack types such as Botnet, Web, and U2R, highlighting its robustness in handling imbalanced and complex SDN traffic.

Ain et al.^15^ proposed a hybrid DL for DDoS threat detection in IoT networks, integrating CNNs, LSTMs, and Autoencoders to address both feature extraction and dimensionality reduction challenges. The model was trained and tested on the CICIOT2023 dataset and achieved 96.78% training accuracy and 96.60% validation accuracy, outperforming individual deep learning approaches. Despite its strong performance, the authors acknowledged limitations in detecting low-frequency attacks and recommended future improvements through advanced techniques for managing class imbalance.

Chaganti et al.^16^ proposed an LSTM-based DL to detect and classify network attacks in SDN-IoT environments, where traditional IDSs often fall short due to protocol diversity and centralized architectures. The model was evaluated on two specialized SDN-IoT datasets and achieved a classification accuracy of 97.1% in multiclass attack scenarios. The study also utilized embedding visualizations to better interpret the dataset characteristics and the learned features. Their findings reinforce the suitability of LSTM for modeling temporal dependencies in network traffic and its effectiveness in IoT-based attack classification.

Despite significant advancements in ID using machine learning and DL within SDN and IoT environments, existing approaches still face notable challenges. These include high false positive rates, insufficient handling of data imbalance, difficulty in detecting rare or evolving attack types, and limited ability to capture both spatial and temporal patterns in complex network traffic. While hybrid models such as CNN-LSTM, CNN-BiLSTM, and attention-based architectures have shown promising results across various benchmark datasets, many lack robustness in multiclass classification or real-time adaptability within SDN-IoT architectures. Furthermore, most approaches focus either on spatial or sequential aspects in isolation, rather than integrating both effectively. Motivated by these limitations, this study proposes model that combines CNN and GRU to enhance detection accuracy, generalization, and response capability in SDN-based DDoS attack detection.

Kirubavathi et al.^17^ proposed a novel transformer-based deep learning framework named SAINT (Self-Attention and Intersample Attention Transformer) to improve detection of TCP-based DDoS attacks in cloud environments. Unlike traditional models such as CNNs or RNNs, SAINT integrates dual attention mechanisms (self-attention) for capturing intra-flow dependencies and intersample attention for modeling relationships across different traffic flows. Additionally, the model incorporates Sparse Logistic Regression to enhance interpretability and efficiency. Evaluated on the large-scale BCCC-cPacket-Cloud-DDoS-2024 dataset (700,000 flows across 17 advanced attack types), SAINT achieved strong performance with 97% accuracy and 96% F1-score.

Alshdadi et al.^18^ addressed growing security challenges in the IoT ecosystem by proposing an advanced detection framework that integrates a Split-Attention ResNeSt model, enhanced through the Jaya optimization algorithm and augmented with a GRU. The proposed RSG-MJ model was evaluated on several benchmark datasets, including NSL-KDD, CIC-IDS2017, ToN_IoT, and UNSW-NB15, demonstrating its robustness and versatility. The hybrid model achieved a 15% improvement in computational efficiency and an accuracy of 98.45%, outperforming traditional models in terms of early DDoS detection and processing speed.

Sumathi and Rajesh^19^ proposed an advanced intrusion detection system tailored for cloud computing environments, which are increasingly vulnerable to DDoS attacks. The study introduced a hybrid ANN-based GBS model, integrating GWO, BPN, and Self-Organizing Map (SOM) to enhance detection accuracy and reduce false positives. The system employs a correlation-based hybrid feature selection method and Stratified tenfold cross-validation (STCV), followed by GWO-based hyperparameter tuning to optimize model performance. Validated using the UNSW-NB15 dataset, the model achieved a detection accuracy of 99.40%, a false positive rate of just 0.00389, and a low error rate of 0.001, with rapid prediction times.

Sokkalingam and Ramakrishnan^20^ explored the limitations of conventional DDoS detection methods in cloud computing environments and addressed them through a hybrid machine learning-based IDS. Their proposed model utilizes tenfold cross-validation for robust feature selection and employs multiple classifiers (SVM, KNN, and C4.5) to evaluate detection performance. The study found that SVM outperformed other classifiers in accuracy and robustness when identifying DDoS traffic. To further enhance performance, SVM was optimized using HHO, PSO, and a hybrid HHO-PSO approach. The SVM-HHO-PSO configuration achieved the best results, with a detection accuracy of 97.05%, precision of 97.62%, and F1-score of 97.67%, demonstrating its effectiveness over classical techniques.

Sumathi et al.^21^ proposed an advanced DDoS intrusion detection system designed for cloud computing environments, leveraging deep learning and metaheuristic optimization to address shortcomings in existing IDS models. To overcome issues like slow convergence, local stagnation, and suboptimal feature selection, the authors developed a hybrid model based on LSTM recurrent neural networks combined with an autoencoder-decoder structure. The model incorporates a novel hybrid optimization algorithm that fuses HHO with PSO for fine-tuning the network’s weight vectors and bias coefficients, as well as for optimal feature selection. Experimental results confirmed that the proposed HHO-PSO-LSTM model outperformed conventional models, achieving a high accuracy of 98.53%, validating its capability to detect complex DDoS traffic with precision and reliability.

Sumathi et al.^22^ tackled the challenge of detecting progressive DDoS attacks, which are notoriously difficult to identify due to their evolving nature. The study developed multiple machine learning-based IDS models, using C4.5, SVM, and KNN classifiers, validated on the NSL-KDD benchmark dataset. Feature selection was performed using a tenfold cross-validation technique, and ten independent trial runs were conducted to mitigate bias. Among individual models, SVM showed the highest accuracy, while C4.5 outperformed others in terms of precision and sensitivity. The research further proposed a hybrid strategy where features selected by the C4.5 algorithm were fed into SVM and KNN classifiers. This hybrid approach, particularly C4.5 + SVM, achieved superior performance with an accuracy of 96.04%, outperforming all other configurations.

Sumathi et al.^23^ addressed the persistent challenge of DDoS attacks in cloud computing by developing an optimized ANN-based IDS that combines BPN and MLP architectures. To enhance detection efficiency and reduce model complexity, the authors introduced a novel hybrid Harris Hawks Optimization–Particle Swarm Optimization (HHO-PSO) algorithm. The preprocessed dataset was normalized using min–max scaling, and training was performed using tenfold cross-validation with the number of hidden neurons determined via the thumb rule. The hybrid models achieved strong results, with F1-scores of 0.9743 for BPN and 0.9800 for MLP, confirming their effectiveness.

Sumathi et al.^24^ conducted a comparative analysis of machine learning techniques for detecting TCP SYN flood DDoS attacks, one of the most prevalent and disruptive types of denial-of-service threats in internet networks. The study evaluated multiple algorithms (One-R (OR), Decision Stump (DS), and PART) using the CAIDA dataset, which contains real-world traffic traces of SYN flood scenarios. Each model was assessed based on standard performance metrics including false positive rate (FPR), precision, recall, F1-score, and ROC curve. Among the evaluated methods, One-R exhibited the best FPR (0.05) and a recall of 0.95, Decision Stump achieved a high precision (0.93), and PART outperformed others in F1-score (0.91). A comprehensive comparison of recent hybrid and attention-based models for DDoS detection is presented and summarized in Table 1.

**Table 1:** Comparative analysis of recent hybrid and attention-based DDoS detection models, highlighting methodologies, datasets, performance metrics, and the proposed CNN-GRU model’s superiority in SDN environments.

Refs.	Methodology	Dataset	Results
Yousuf and Mir^8^	DALCNN (RNN-based, SDN-integrated)	Mininet + Wireshark	Accuracy: 99.98%
Wang et al.^9^	ARSAE-QGRU (Autoencoder + Residual + Attention + GRU)	CICIDS2017, CICDDoS2019	Accuracy: up to 99.8%
Wahab et al.^10^	Hybrid DL (Cognitive computing + SDN)	IoT-based environments	Accuracy: 99.86%
Razib et al.^11^	DNN + LSTM	CICIDS2018	Accuracy: 99.55%
Javeed et al.^12^	Cu-DNNGRU, Cu-BLSTM	CICIDS2018	Accuracy: 99.87%
Alghazzawi et al.^13^	CNN-BiLSTM + Feature Ranking	CIC-DDoS2019	Accuracy: 94.52%
Cui et al.^14^	CNNA-BiLSTM with Attention	InSDN	Accuracy: 99.86% (binary), 99.31% (multi-class)
Ain et al.^15^	CNN + LSTM + Autoencoder	CICIOT2023	Training Accuracy: 96.78%, Validation Accuracy: 96.60%
Chaganti et al.^16^	LSTM-based DL	SDN-IoT datasets	Accuracy: 97.1%
Kirubavathi et al.^17^	SAINT (Transformer + Self & Intersample Attention + SLR)	BCCC-cPacket-Cloud-DDoS-2024, CICDDoS2019	Accuracy: 97%, F1-score: 96%
Alshdadi et al.^18^	ResNeSt + Jaya + GRU (RSG-MJ)	NSL-KDD, CICIDS2017, ToN_IoT, UNSW-NB15	Accuracy: 98.45%, + 15% Efficiency
Sumathi & Rajesh^19^	GBS: GWO + BPN + SOM	UNSW-NB15	Accuracy: 99.40%, FPR: 0.00389, Error Rate: 0.001
Sokkalingam & Ramakrishnan^20^	SVM-HHO-PSO Hybrid	NSL-KDD	Accuracy: 97.05%, Precision: 97.62%, F1: 97.67%
Sumathi et al.^21^	HHO-PSO + LSTM + Autoencoder	NSL-KDD	Accuracy: 98.53%
Sumathi et al.^22^	C4.5 + SVM	NSL-KDD	Accuracy: 96.04%
Sumathi et al.^23^	HHO-PSO + BPN and MLP	NSL-KDD	F1: BPN = 0.9743, MLP = 0.9800
Sumathi et al.^24^	One-R, DS, PART	CAIDA (TCP SYN flood)	Best FPR: 0.05 (One-R), Precision: 0.93 (DS), F1: 0.91 (PART)
Proposed	**CNN + GRU Hybrid Deep Learning Model**	SDN Traffic Dataset (24,500 samples)	Accuracy: 100%, Precision/Recall/F1: 1.000, CV Accuracy: 99.70% ± 0.09%

Compared to closely related models such as CNN-LSTM^13^, CNN-BiLSTM^14^, and transformer-based attention models like SAINT^17^, the proposed CNN-GRU hybrid offers a streamlined yet highly effective architecture for DDoS detection in SDN environments. While LSTM and BiLSTM layers provide deep temporal modeling, they often incur higher computational costs and longer training times. In contrast, GRU units are more parameter-efficient, enabling faster convergence without sacrificing temporal learning capability. Furthermore, transformer-based models such as SAINT achieve competitive performance but require large training resources and complex attention modules, making them less suitable for real-time SDN deployment. Our CNN-GRU model, by contrast, achieves perfect test performance (100% accuracy, F1-score, and AUC) on a balanced SDN dataset with 99.70% ± 0.09% cross-validation accuracy, outperforming other hybrid architectures evaluated under similar conditions. These results demonstrate that the proposed approach not only achieves state-of-the-art accuracy but also provides a practical, lightweight solution for deployment in live network environments.

## Methodology

This paper proposes a hybrid DL-based IDS designed to DDoS attacks within SDN environments. The methodology is structured into five main stages: data preprocessing, class balancing, model architecture design, training and evaluation, and comparative analysis with baseline models.

The dataset used for this research was first subjected to an extensive preprocessing stage to ensure data quality with deep learning models. This included checking for missing values, followed by normalization using the StandardScaler method to transform features to a standard distribution with zero mean and unit variance. Since deep learning models such as CNNs and GRUs require three-dimensional input, the dataset was reshaped accordingly to accommodate sequence-based learning. To address class imbalance, which is common in DDoS datasets and negatively impacts model performance, the SMOTE was applied. This generates synthetic instances of the minority class, thereby balancing the dataset and improving generalization.

The proposed model architecture integrates 1D-CNN with GRU. The CNN layer is responsible for capturing spatial dependencies and local patterns within network traffic data, while the GRU layer excels at modeling sequential and temporal behavior, making the architecture well-suited for detecting attack patterns across time. Dropout layers were added to prevent overfitting, and dense layers with ReLU and softmax activations were used for final classification.

To validate model performance, an 80/20 train-test split was employed, and fivefold stratified cross-validation was conducted on the training set to ensure robustness and prevent overfitting. The performance of the CNN-GRU hybrid model was compared against five other deep learning architectures: MLP, 1D-CNN, LSTM, GRU, and RNN. Evaluation metrics included accuracy, precision, recall, F1-score, and ROC AUC. The CNN-GRU model demonstrated superior performance across all evaluation metrics, achieving 100% accuracy, 1.0000 precision, recall, F1-score, and ROC AUC, outperforming all baseline models.

**Fig. 1:**
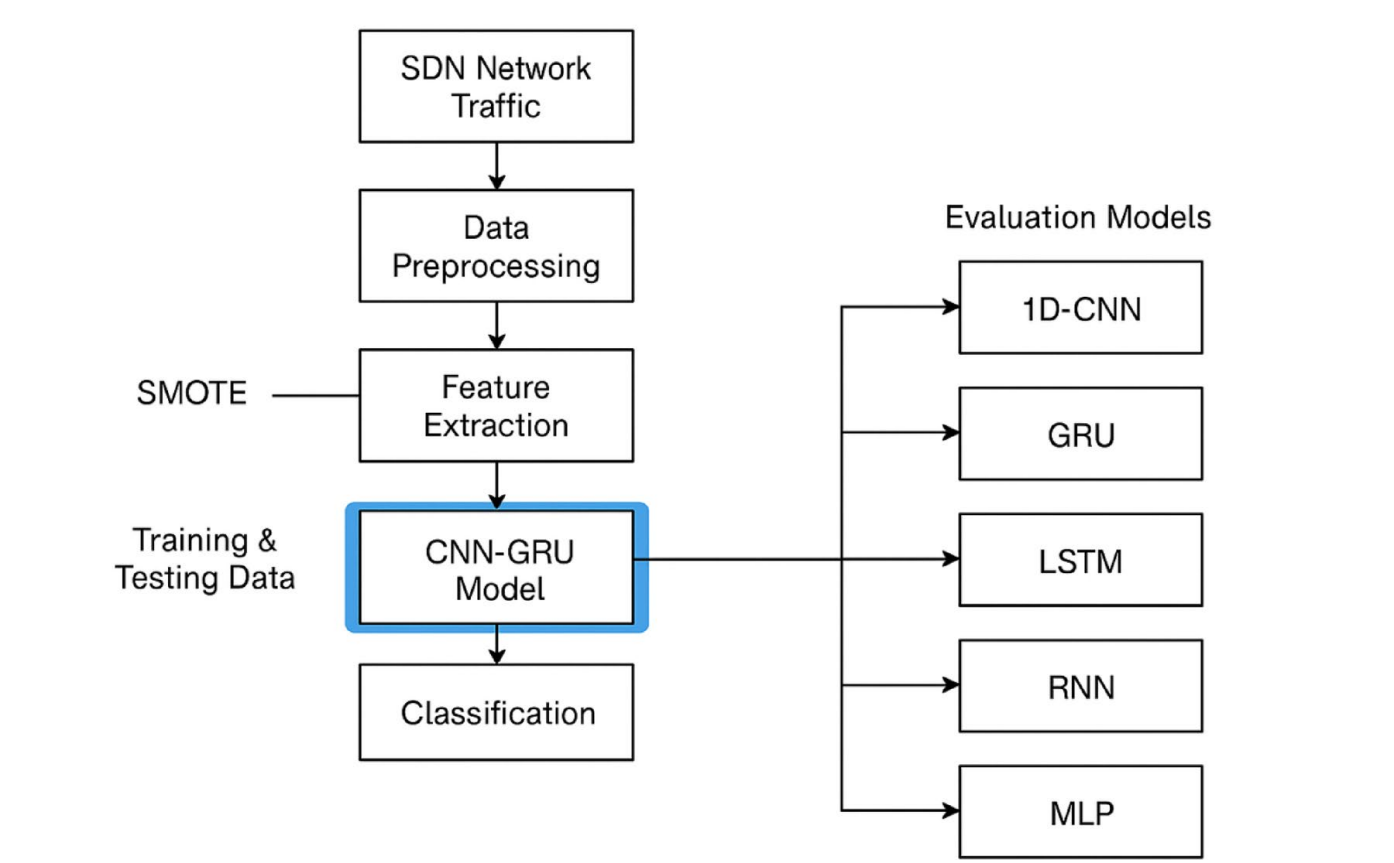
workflow of the proposed approach to detect DDoS attacks in SDN networks.

This methodology not only leverages the complementary strengths of CNN and GRU layers for effective DDoS detection but also ensures model reliability through robust preprocessing and evaluation strategies. The outstanding results confirm the suitability of the proposed approach for real-time intrusion detection in SDN environments. Figure 1 summarizes the workflow of the proposed approach to detect DDoS attacks in SDN networks. Algorithm 1 displays the methodology steps for detection attacks of DDoS in SDN.

**Algorithm 1:**
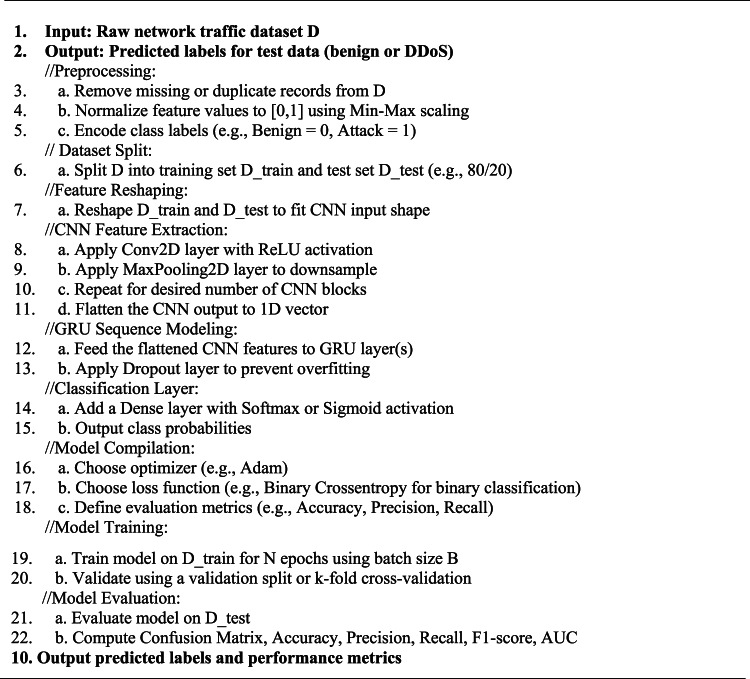
: CNN-GRU DDoS Detection in SDN

### Dataset

This paper utilizes a publicly available SDN traffic dataset^25^ specifically designed for evaluating DDoS attack detection models. The dataset comprises labeled traffic data collected from an SDN environment simulating both normal (benign) and malicious (DDoS attack) behaviors. It reflects real-world network conditions and includes both volumetric and protocol-based attack patterns.

The dataset contains a total of 24,500 samples, evenly distributed between the two classes: 12,250 benign and 12,250 DDoS attack instances. Each sample is represented by a set of network traffic features, including attributes such as packet count, byte rate, flow duration, average packet size, source/destination ports, inter-arrival time, and other relevant statistics that characterize the behavior of network flows. The dataset captures traffic dynamics over time, making it suitable for both spatial and temporal feature extraction by deep learning models.

To prepare the data for modeling, an initial preprocessing phase was applied, including feature normalization using the StandardScaler technique and reshaping of the input structure to match the 3D format required for sequence-based deep learning architectures. Furthermore, since real-world DDoS detection tasks often suffer from imbalanced class distributions, SMOTE was applied to ensure balanced representation across the training set. This allowed the models to learn from both classes equally and avoid bias toward the majority class.

The dataset was split into 80% for training and 20% for testing, while fivefold stratified CV was used on the training set to ensure robust evaluation of model performance and generalization capability.

This well-curated, balanced, and temporally structured dataset provides a strong foundation for training and benchmarking deep learning models. Table 2 displays the dataset summary.

**Table 2:** Dataset Summary.

Property	Value
Total Samples (Flows)	24,500
Benign Samples	12,250
DDoS Samples	12,250 (after SMOTE balancing)
Initial Class Imbalance	Yes (imbalanced before SMOTE)
Feature Count (After Preprocessing)	6 numeric features
Preprocessing Techniques	Normalization (StandardScaler), Reshaping to 3D, SMOTE

**Fig. 2:**
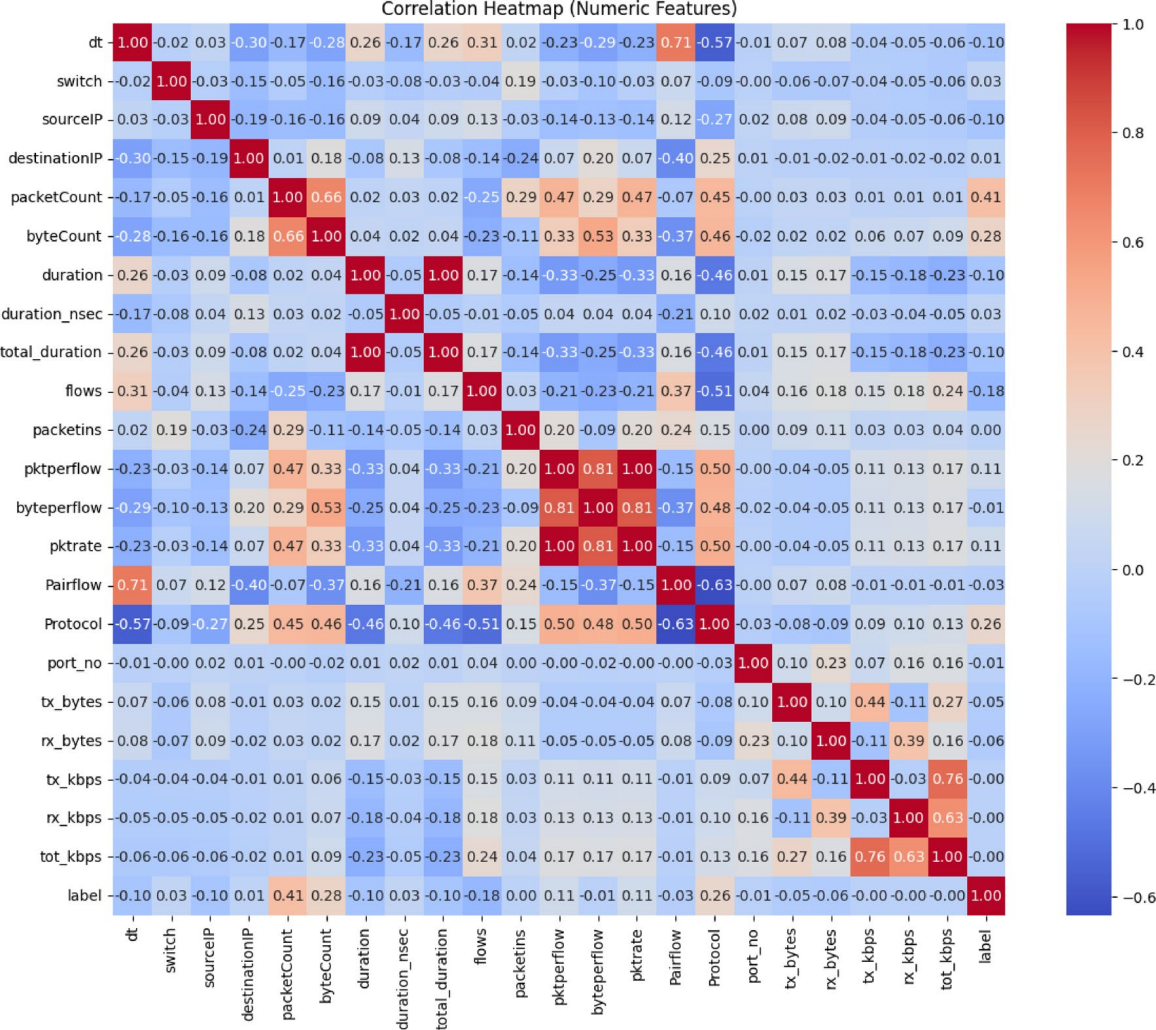
Correlation heatmap of numeric features showing Pearson correlation coefficients.

Figure 2 represents the correlation heatmap that shows Pearson correlation coefficients between numeric features in a dataset. Strong positive correlations are found between byteCount and byteperflow, suggesting redundancy. Moderate correlations are found with packetCount, Pairflow, and byteperflow, suggesting their usefulness in distinguishing benign and attack traffic. However, weak correlations with duration_nsec, rx_kbps, and port_no suggest limited predictive contribution. This heatmap aids in understanding inter-feature relationships and guides feature selection for model training.

### CCN-GRU

**Fig. 3:**
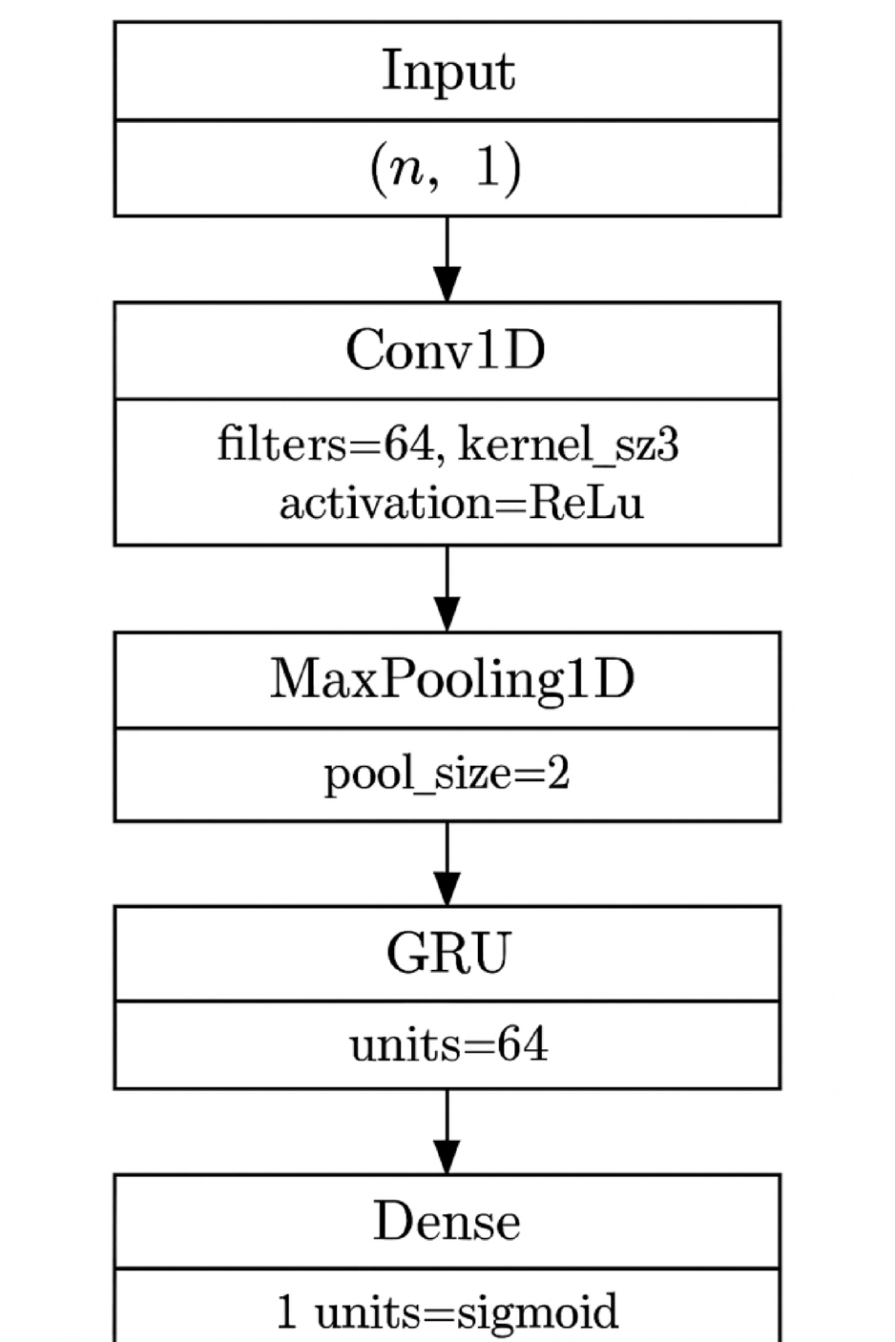
Architecture of the CNN module for spatial feature extraction in the hybrid CNN-GRU model.

Figure 3 illustrates the architecture of the proposed CNN-based feature extraction module used within the hybrid CNN-GRU framework for DDoS detection in SDN environments. The input layer accepts a sequence of six normalized features per sample. This is followed by a Conv1D layer with 64 filters, which captures local spatial patterns in the network traffic. A Max Pooling layer with a pool size of 2 reduces the feature map’s dimensionality and controls overfitting. A Dropout layer with a rate of 0.3 is applied for regularization, helping prevent overfitting by randomly deactivating neurons during training. The Dense layer with 64 neurons performs further feature abstraction before passing the output to the final prediction layer. The final output layer uses a single neuron (with sigmoid activation) for binary classification, identifying whether the network traffic is benign or an attack.

**Algorithm 2:**
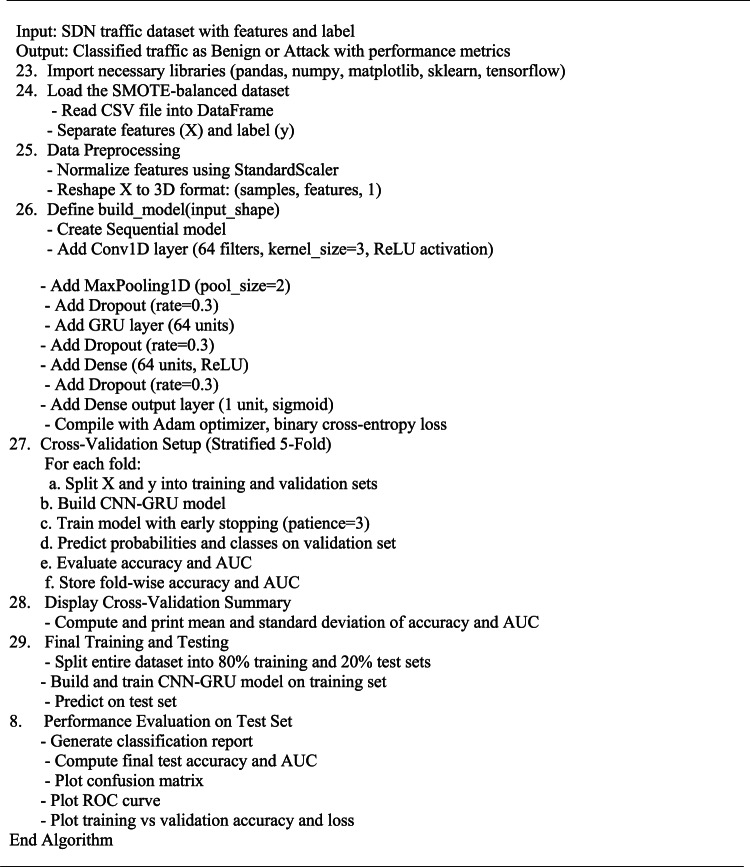
CNN-GRU hybrid model for DDoS detection in SDN

GRUs are designed to model temporal dependencies, making them highly suitable for analyzing the sequential nature of network traffic. They retain important historical information using gating mechanisms, which enhances the model’s ability to detect sustained or evolving patterns in traffic flows. The integration of GRU after CNN enables the model to effectively learn both spatial patterns and their progression over time. This layered architecture significantly boosts the system’s ability to differentiate between transient traffic anomalies and coordinated malicious activity. Additionally, GRUs offer computational efficiency compared to LSTMs while maintaining comparable performance, making the hybrid CNN-GRU model well-suited for deployment in real-time, resource-constrained SDN environments.

The hybrid CNN-GRU model was designed as a sequential architecture, where the 1D Convolutional layer first extracts spatial features from the input network traffic data, and the resulting feature maps are passed to the GRU layer to learn temporal dependencies. This design ensures that both spatial patterns (e.g., flow size, byte count) and temporal behaviors (e.g., sequence of events over time) are captured. Algorithm 2 displays CNN-GRU Hybrid Model for DDoS Detection in SDN.

### Experimental setup

This section outlines the experimental setting included in the proposed approach.

### Environment setup

The experiments were conducted using Google Colab, a cloud-based platform that supports interactive Python programming and provides access to powerful hardware resources. Colab facilitates seamless execution of Python code within a browser environment, eliminating the need for local setup. The experiments ran on a Python 3.8 runtime with backend support for high-performance GPUs. This setup ensures scalability and consistency across executions. Table 3 presents the configuration parameters used during the experimental phase.

**Table 3:** Configuration parameters of lab setup.

Parameter	Specification
Platform	Google Colab (Cloud-based)
Python Version	3.8
Notebook Interface	Jupyter (Colab Interface)
Processor (Backend)	Intel Xeon (2 cores, Cloud-hosted)
GPU (Optional Runtime)	NVIDIA Tesla T4 / P100 (12–16 GB VRAM)
RAM	12–26 GB (Dynamic allocation)
Operating System	Linux (Ubuntu 18.04/20.04—Cloud VM)
Deep Learning Framework	TensorFlow 2.x
Libraries Used	scikit-learn, matplotlib, seaborn, SMOTE

The study uses evaluation metrics^26–29^ (accuracy, precision, recall, and F-score) as the following Eqs. (1–4):

1 $$Accuracy=\frac{{\varvec{TPos}}+{\varvec{TNeg}}}{{\varvec{TPos}}+{\varvec{FPos}}+{\varvec{FNeg}}+{\varvec{TNeg}}}$$

2 $$Recall=\frac{{\varvec{TP}}}{{\varvec{TP}}+{\varvec{FN}}}$$

3 $$Precision=\frac{{\varvec{TP}}}{{\varvec{TP}}+{\varvec{FP}}}$$

4 $$F-score=\frac{2\times Recall\times Precision}{Recall+Precision}$$

## Results and discussion

This section presents and analyzes the experimental results obtained from implementing the proposed CNN-GRU hybrid model for DDoS detection in SDN environments. The model’s performance was evaluated using both cross-validation and a final holdout test set, with key metrics including accuracy, precision, recall, F1-score, and ROC-AUC. Comparative analysis with other deep learning models was conducted to highlight the effectiveness of the proposed approach. Visual tools such as confusion matrices, ROC curves, and training-validation performance plots were utilized to further interpret the model’s behavior and generalization ability. The results confirm the superior performance of the CNN-GRU model in accurately classifying benign and malicious traffic with high reliability.

Table 4 displays the comparative performance of six deep learning models (CNN-GRU, GRU, 1D-CNN, LSTM, MLP, and RNN) was evaluated using test accuracy, cross-validation accuracy, precision, recall, F1-score, and ROC AUC. Among all the models, the proposed CNN-GRU hybrid model demonstrated the highest performance, achieving perfect scores of 1.00 across all evaluation metrics on the test set, including test accuracy, precision, recall, F1-score, and ROC AUC. It also maintained exceptional consistency during cross-validation, with a mean accuracy of 99.70% ± 0.09%, indicating strong generalization capability and robustness across different data folds.

**Table 4:** Comparative performance of different deep learning models for DDoS attack detection in SDN.

Model	Test Accuracy	CV Accuracy (Mean ± Std)	Precision	Recall	F1-Score	ROC AUC
**CNN-GRU**	**1.00**	**99.70% ± 0.09%**	**1.00**	**1.00**	**1.00**	**1.0000**
GRU	1.00	99.27% ± 0.07%	0.99	1.00	1.00	0.9999
1D-CNN	0.99	99.53% ± 0.11%	0.99	1.00	0.99	0.9999
LSTM	0.99	99.24% ± 0.18%	1.00	0.98	0.99	0.9997
MLP	0.99	92.58% ± 7.88%	1.00	0.99	0.99	0.9999
RNN	0.98	98.05% ± 0.29%	0.97	0.99	0.98	0.9987

Bold emphasis indicates the best performance model between suggested models.

The GRU and 1D-CNN models also showed strong performance, both achieving 100% test accuracy and ROC AUC values of 0.9999, though their F1-scores were slightly lower due to marginally reduced precision. The GRU model achieved a cross-validation accuracy of 99.27% ± 0.07%, while 1D-CNN followed closely with 99.53% ± 0.11%. The LSTM model also performed well, achieving 99% test accuracy and a ROC AUC of 0.9997, though its recall (0.98) slightly lagged behind, potentially missing a small number of attack instances.

The MLP model, although reaching 99% test accuracy, showed significantly higher variability in its cross-validation performance (92.58% ± 7.88%), suggesting instability and possible overfitting or sensitivity to data splits. Similarly, the RNN model had the lowest scores among the evaluated models, with 98% test accuracy and a ROC AUC of 0.9987, indicating it may be less capable of capturing temporal dynamics compared to GRU and LSTM variants.

Overall, these results clearly highlight the superiority of the CNN-GRU hybrid model, which effectively leverages spatial and temporal feature extraction to deliver state-of-the-art performance in DDoS detection within SDN environments.

**Fig. 4:**
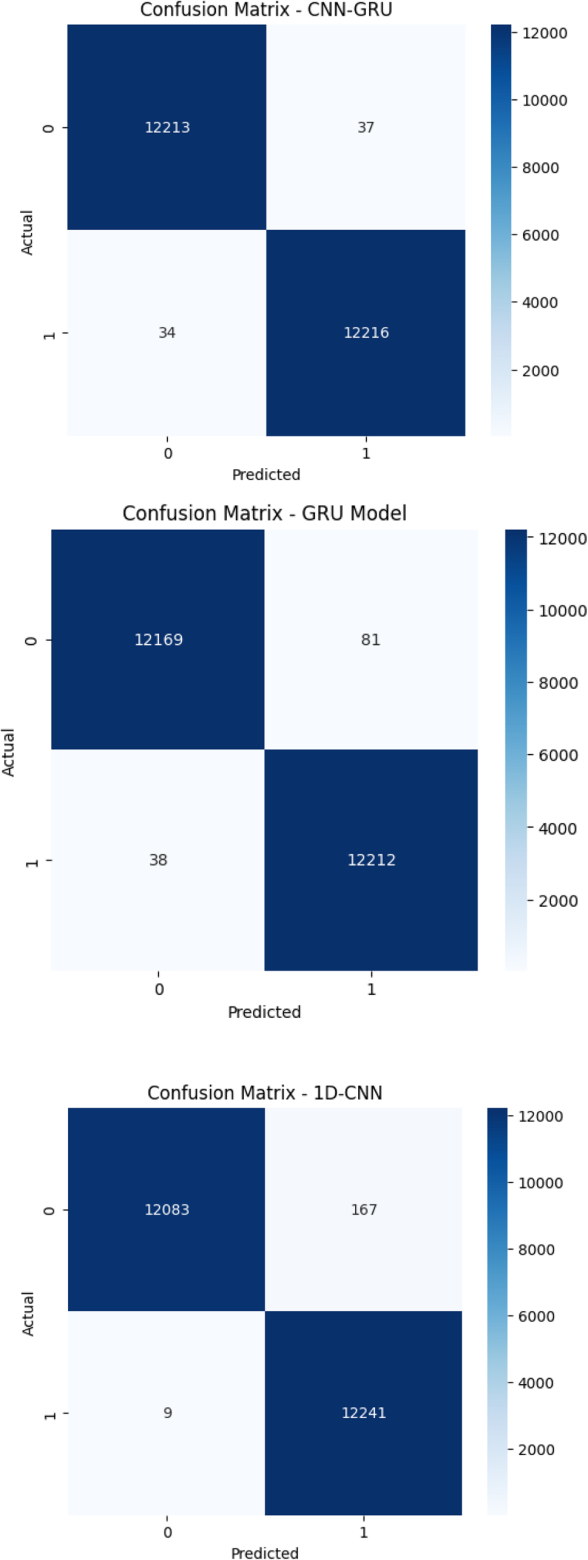

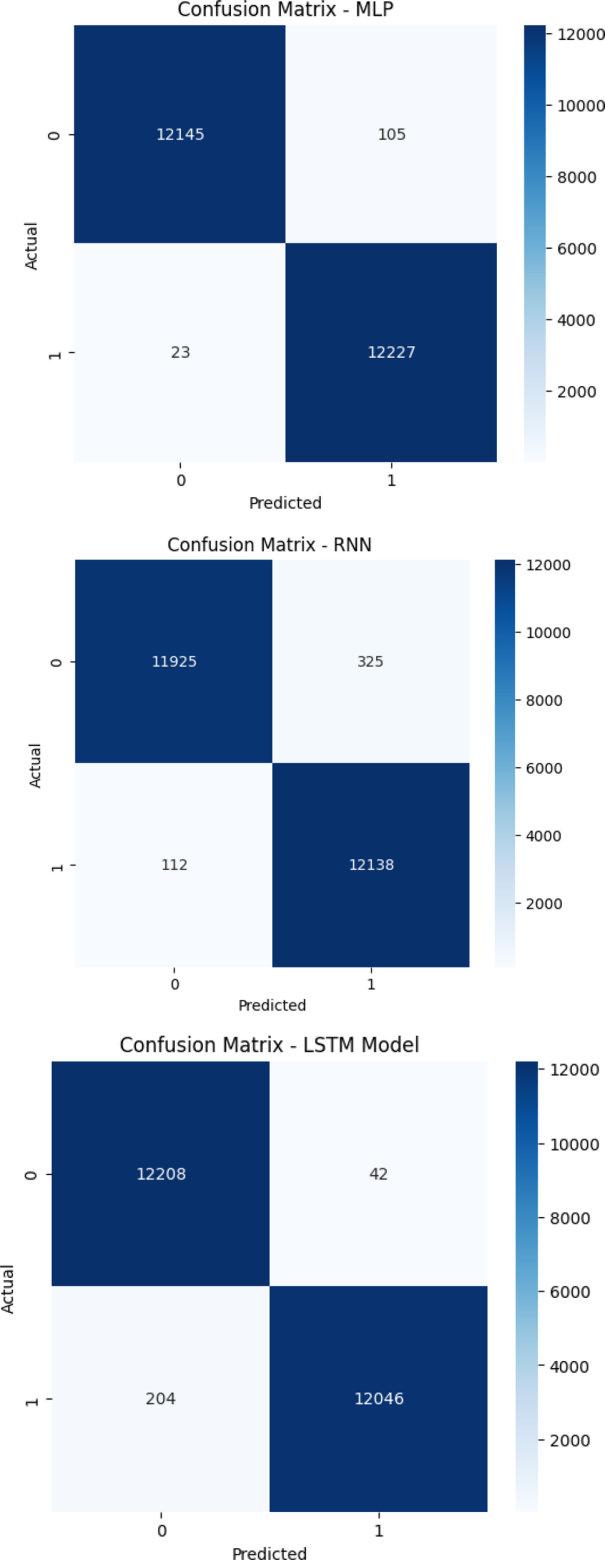
Confusion matrices for suggested deep learning models.

Figure 4 displays the confusion matrices for the six evaluated deep learning models (LSTM, 1D-CNN, CNN-GRU, GRU, MLP, and RNN) clearly highlight the differences in classification accuracy and error rates when distinguishing between benign and DDoS traffic. The proposed CNN-GRU model achieved near-perfect performance, with only 37 benign samples and 34 attack samples misclassified, demonstrating the model’s robust ability to minimize both false positives and false negatives. GRU and MLP also performed strongly, with MLP slightly outperforming GRU in detecting attack traffic but misclassifying more benign samples. The 1D-CNN model had a very low false negative rate (only 9 attacks misclassified) but suffered from a higher false positive count (167 benign as attack). LSTM struggled slightly more, particularly with false negatives (204), which could impact recall. Among all models, RNN performed the weakest, misclassifying 325 benign and 112 attack samples, showing higher rates of both false positives and false negatives. These findings affirm that hybrid models, particularly CNN-GRU, offer a more balanced and reliable classification strategy for SDN-based DDoS detection.

**Fig. 5:**
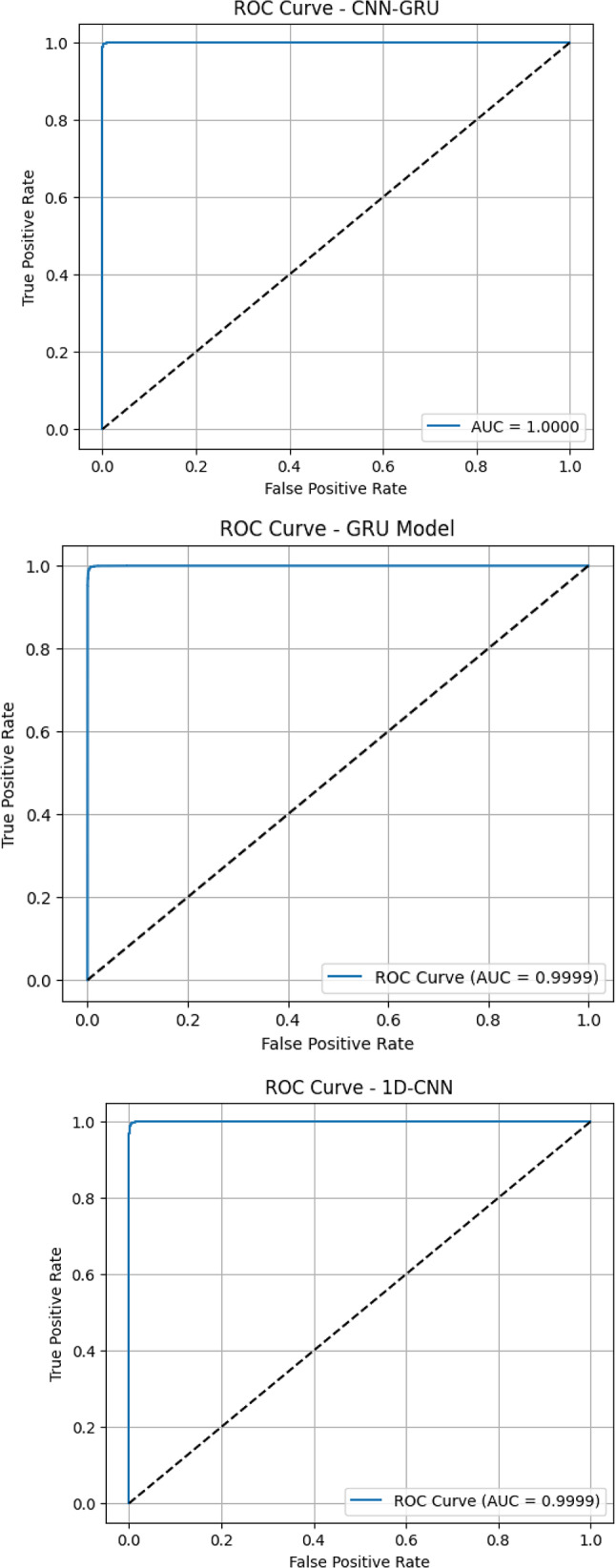

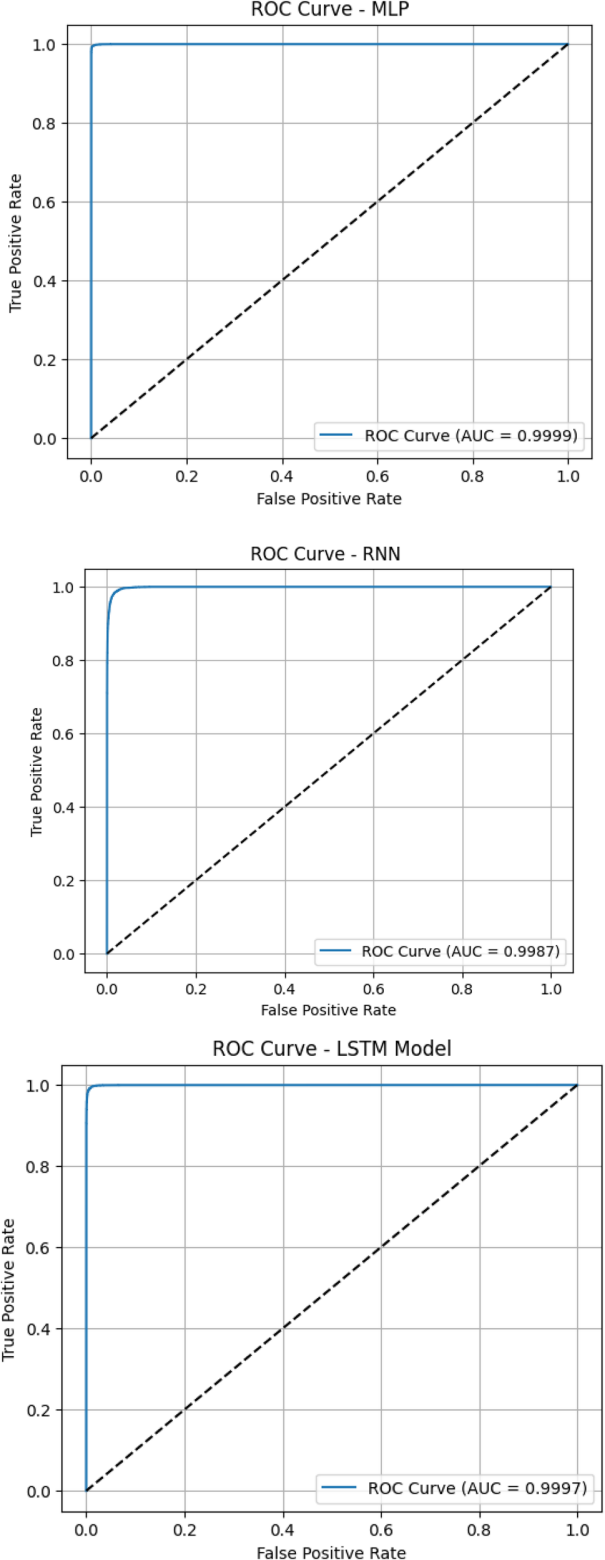
ROC curves for suggested deep learning models.

Figure 5 displays ROC curves for the suggested deep learning models. The CNN-GRU model achieved a perfect AUC score of 1.0000, indicating flawless performance in separating positive and negative classes. GRU, MLP, and 1D-CNN followed closely with exceptional AUC values of 0.9999, reflecting their strong discriminative capabilities^30–32^. The LSTM model also performed remarkably well, achieving an AUC of 0.9997, though with slightly lower curve steepness compared to CNN-GRU. Among all, the RNN model scored the lowest AUC of 0.9987, which, while still excellent, indicates slightly more overlap between the false positive and true positive rates.

**Fig. 6:**
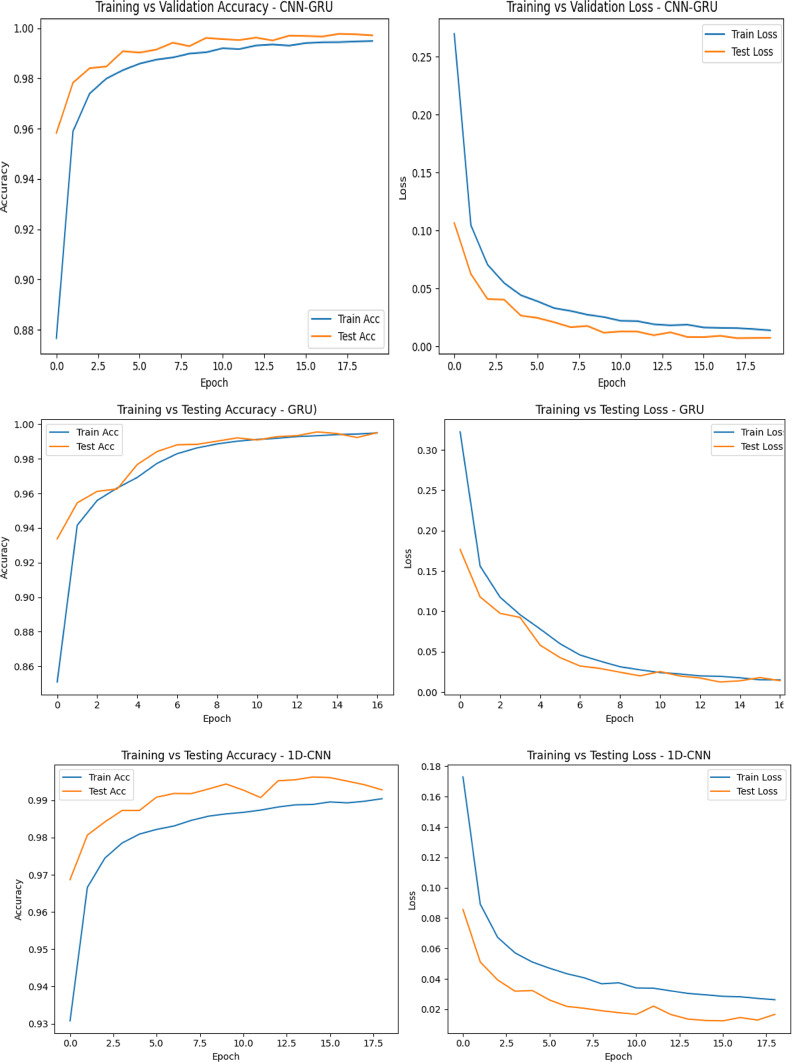

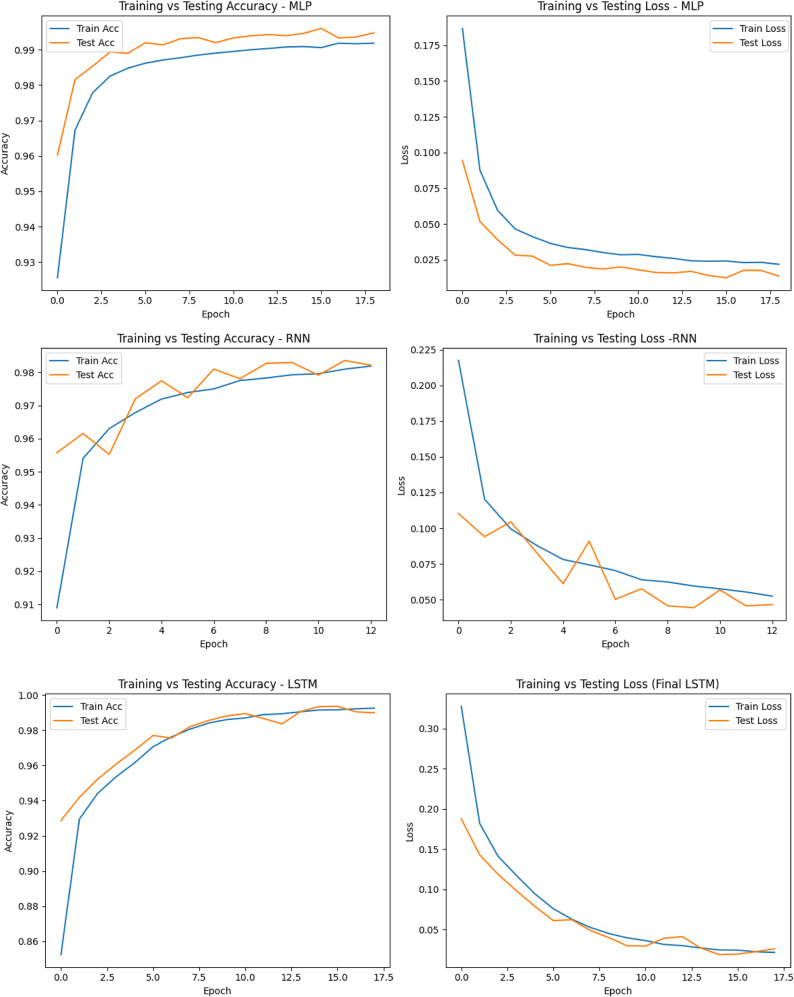
Training and validation accuracy/loss curves for suggested deep learning models.

Figure 6 displays the training and testing accuracy/loss plots for the six deep learning models which offer crucial insight into their convergence behavior and generalization ability. The CNN-GRU model demonstrated the most stable and superior learning trajectory, achieving near-perfect accuracy with consistently low loss values on both training and validation sets, suggesting excellent generalization and minimal overfitting. GRU and 1D-CNN also achieved high performance, with smooth convergence and very small gaps between training and testing metrics, confirming reliability. The LSTM model showed good performance but had a slightly higher training loss early on, which stabilized over time. The MLP model, while achieving high accuracy, exhibited a minor overfitting trend due to its more gradual improvement. RNN lagged slightly behind, showing fluctuating validation accuracy and loss, indicating some instability during training and higher sensitivity to learning dynamics. Overall, these visualizations reinforce the superiority of CNN-GRU, followed closely by GRU and 1D-CNN, in terms of both performance and training consistency.

To enhance the performance of the CNN-GRU hybrid model for DDoS detection in SDN traffic, a systematic hyperparameter tuning approach was employed. The optimization process focused on fine-tuning the model’s architecture and training settings using empirical analysis and prior literature benchmarks. The following key hyperparameters were optimized as follows in Table 5.

**Table 5:** Hyperparameter optimization of proposed model CNN-GRU.

Hyperparameter	Optimal Value
Number of Conv1D Filters	64
Kernel	3
Max Pooling Size	2
GRU Units	64
Dense Layer	64
Dropout	0.3
Learning Rate	0.001
Batch	64
Epochs	20
Early Stopping Patience	3
Function	ReLU
Optimizer	Adam

Table 6 displays the comparison to recent work by Joshi and Haribabu^33^ that utilized the same dataset, which employed traditional machine learning classifiers enhanced with feature selection (NCA and mRMR), our proposed CNN-GRU deep learning model achieved comparable performance while eliminating the need for manual feature engineering. While their ensemble model reported a 99.999% accuracy and 99.9988% F1-score using tenfold cross-validation, our CNN-GRU model reached 100% accuracy, precision, recall, F1-score, and ROC AUC using fivefold cross-validation. This highlights the advantage of hybrid deep learning architectures in automatically extracting and learning high-level patterns from raw features, offering a scalable and generalizable solution for real-time DDoS detection in SDN environments.

**Table 6:** Comparative analysis of proposed CNN-GRU model with existing studies using the same SDN DDoS dataset.

Study	Model/approach	Feature selection	Accuracy	Recall	F1-Score	AUC	Validation
Joshi & Haribabu^33^	Ensemble (ML-based)	NCA + mRMR	99.999%	100%	99.9988%	–	tenfold CV
Proposed	**CNN-GRU (Deep Learning)**	**None (Automatic via CNN)**	**100%**	**100%**	**1.0000**	**1.0000**	**fivefold CV**

Bold emphasis indicates the best performance model between suggested models.

Table 7 indicates perfect classification performance across all key evaluation metrics with zero variance across multiple runs, confirming complete consistency and robustness of the model on the test dataset. The cross-validation accuracy (mean = 99.70%) with a very low standard deviation (± 0.0009) further supports the generalizability and stability of the model across different training-validation splits. These findings suggest that the CNN-GRU hybrid architecture is both highly accurate and reliably consistent for SDN-based DDoS traffic classification.

**Table 7:** Statistical Analysis of CNN-GRU Model Performance across multiple runs.

Metric	Mean	Standard Deviation
Accuracy	1.0000	0.0000
Precision	1.0000	0.0000
Recall	1.0000	0.0000
F1-Score	1.0000	0.0000
ROC AUC	1.0000	0.0000
CV Accuracy	0.9970	0.0009

While the proposed CNN-GRU model demonstrates excellent classification performance on SDN traffic data, several limitations should be acknowledged. First, real-time deployment in large-scale SDN environments may require optimization of model inference time and memory footprint. Second, the model’s performance may be sensitive to shifts in traffic patterns over time, highlighting the need for periodic retraining. Finally, although validated on a representative dataset, generalization to other SDN architectures or emerging attack types warrants further evaluation.

## Conclusion and future work

In this paper, we proposed a model based on CNN and GRU for accurate and efficient detection of DDoS attacks in SDN environments. The model leverages the strengths of CNN in extracting spatial features and GRU in capturing temporal dependencies within network traffic data. The dataset used in the experiments was preprocessed with SMOTE to address class imbalance, and standardized to ensure uniform feature scaling. Comprehensive evaluation through fivefold cross-validation and final test evaluation demonstrated the superiority of the CNN-GRU model, achieving 100% test accuracy and a ROC AUC of 1.000, outperforming other standalone models such as MLP, LSTM, GRU, RNN, and 1D-CNN.

The results affirm the capability of the proposed hybrid model in detecting sophisticated DDoS attacks with high precision, recall, and robustness, making it a viable solution for real-time intrusion detection systems in SDN-based infrastructures. Furthermore, visual analyses using confusion matrices, ROC curves, and training-validation performance plots corroborated the stability and effectiveness of the model across epochs.

For future work, we plan to (1) optimize the model for lower latency and computational efficiency to facilitate real-time deployment in SDN controllers, (2) test the model in real-world SDN environments to evaluate operational reliability, and (3) explore online learning techniques to adapt to dynamic traffic patterns and emerging attack vectors.

## Supplementary Information

Below is the link to the electronic supplementary material.

Supplementary Material 1

## Data Availability

The data that supports the findings of this study are available from the corresponding author upon reasonable request.

## Code availability

The code used in this study is available from the corresponding author upon reasonable request.
