# Supplementary material for: DDoS classification of network traffic in software defined networking SDN using a hybrid convolutional and gated recurrent neural network
Source: Sci Rep. 2025 Aug 9;15:29122. doi: 10.1038/s41598-025-13754-1 (PMC12334730; doi:10.1038/s41598-025-13754-1)
Supplement: Supplementary file 1 — Supplementary Material 1 [file 41598_2025_13754_MOESM1_ESM.docx]

**Results on Second Dataset**

To further validate the robustness and generalization capability of the proposed CNN-GRU model, we evaluated its performance on a second DDoS dataset that available at Kaggle <https://www.kaggle.com/datasets/oktayrdeki/ddos-traffic-dataset>. The model achieved exceptional results across all evaluation metrics. Specifically, the cross-validation (CV) process yielded a mean accuracy of 1.0000 with a standard deviation of 0.0000, indicating stable and consistent performance across all folds. On the held-out test set, the model attained a test accuracy of 1.0000, with precision, recall, and F1-score all reaching 1.0000, signifying perfect classification of both benign and attack instances. Furthermore, the ROC AUC score of 1.0000 confirms the model's outstanding ability to discriminate between classes. These results suggest that the CNN-GRU model is highly effective in learning and distinguishing DDoS attack patterns in the given dataset. However, such perfect scores warrant caution, as they may reflect the presence of easily separable patterns or strong predictive features within the dataset. Further evaluations on more diverse and real-world traffic are recommended to confirm generalizability.

Figure 1 displays the confusion matrix for the CNN-GRU model on the second DDoS dataset demonstrates an almost perfect classification performance. Out of 170,517 total instances, the model correctly identified 90,968 benign samples and 79,547 attack samples, with only two benign instances misclassified as attacks and zero false negatives. This results in a false positive rate near zero and a true positive rate of 100%, underscoring the model’s exceptional precision and recall. The minimal error observed highlights the model’s strong discriminative capability in identifying DDoS traffic, suggesting it is highly effective in scenarios where distinguishing between attack and normal traffic is critical.


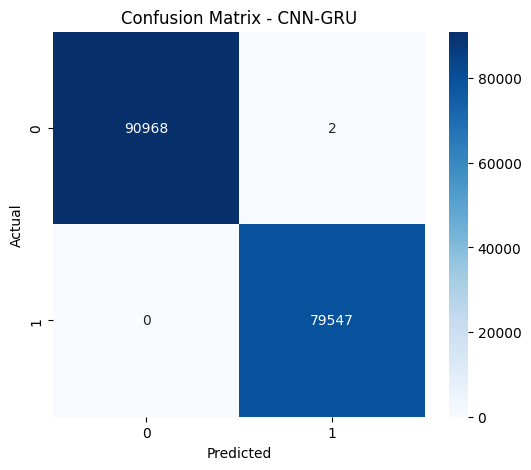


**Figure 1:** Confusion Matrix for the CNN-GRU model on the second DDoS dataset

Figure 2 displays the ROC curve of the CNN-GRU model applied to the second DDoS dataset reveals a near-ideal performance, with the curve hugging the top-left corner of the plot. This indicates that the model successfully distinguishes between benign and attack classes across all classification thresholds. The Area Under the Curve (AUC) is 1.0000, which confirms the model’s perfect sensitivity and specificity. AUC values close to 1 signify that the model has excellent ability to differentiate between positive (attack) and negative (benign) classes without bias, reinforcing the model’s reliability in practical DDoS detection scenarios.


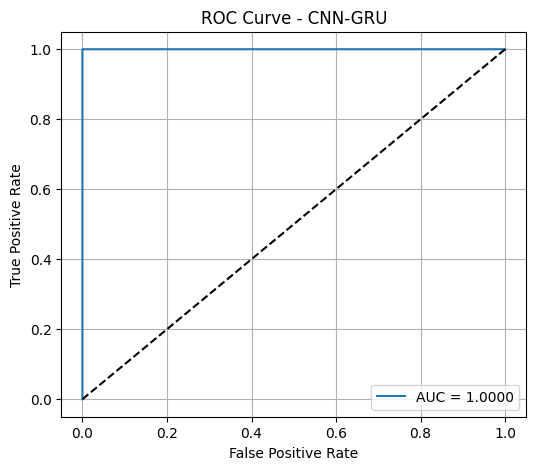


**Figure 2:** ROC Curve of the CNN-GRU model on the second DDoS dataset

Figure 3 displays the training and validation curves for the CNN-GRU model on the second DDoS dataset indicate highly effective and stable learning behavior. The training and validation accuracy both quickly converge to nearly 100% within the first few epochs, reflecting the model’s fast and accurate convergence. Similarly, the loss curves for both training and validation sets decrease sharply and remain close to zero, with minimal gap between them. This convergence pattern validates that the model generalizes well to unseen data while maintaining high learning efficiency throughout training.


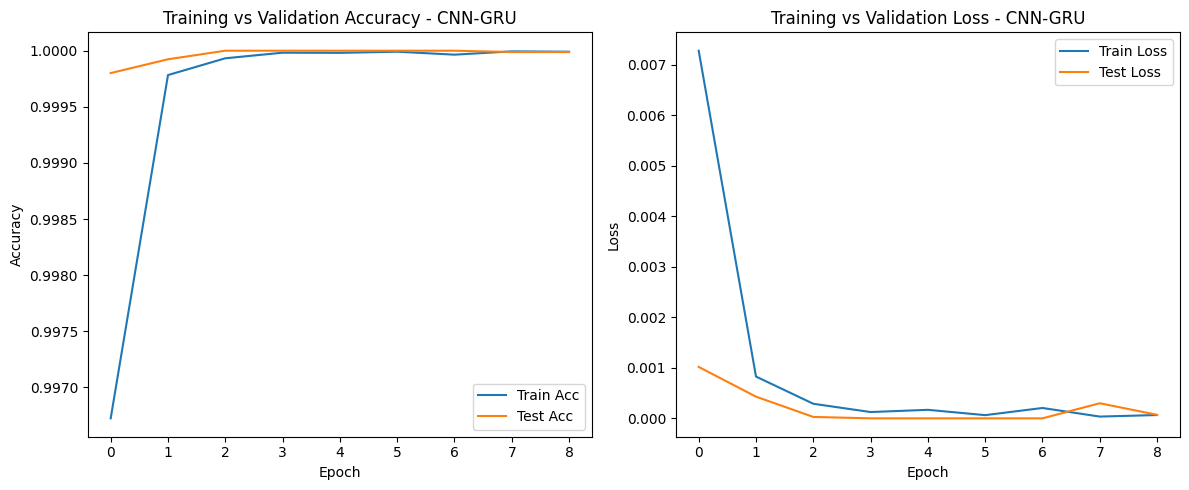


**Figure 3:** Training vs. Validation Accuracy and Loss for the CNN-GRU model on the second DDoS dataset.
